# Improving reproducibility through condition-based sensitivity assessments: application, advancement and prospect†

**DOI:** 10.1039/d4sc03017f

**Published:** 2024-08-30

**Authors:** Felix Schäfer, Lukas Lückemeier, Frank Glorius

**Affiliations:** a Universität Münster, Organisch-Chemisches Institut Corrensstraße 36 48149 Münster Germany glorius@uni-muenster.de

## Abstract

The fluctuating reproducibility of scientific reports presents a well-recognised issue, frequently stemming from insufficient standardisation, transparency and a lack of information in scientific publications. Consequently, the incorporation of newly developed synthetic methods into practical applications often occurs at a slow rate. In recent years, various efforts have been made to analyse the sensitivity of chemical methodologies and the variation in quantitative outcome observed across different laboratory environments. For today's chemists, determining the key factors that really matter for a reaction's outcome from all the different aspects of chemical methodology can be a challenging task. In response, we provide a detailed examination and customised recommendations surrounding the sensitivity screen, offering a comprehensive assessment of various strategies and exploring their diverse applications by research groups to improve the practicality of their methodologies.

## Abstract 2

The fluctuating reproducibility of scientific reports presents a well-recognised issue, frequently stemming from insufficient standardisation, transparency and a lack of information in scientific publications.

## Graphical abstract

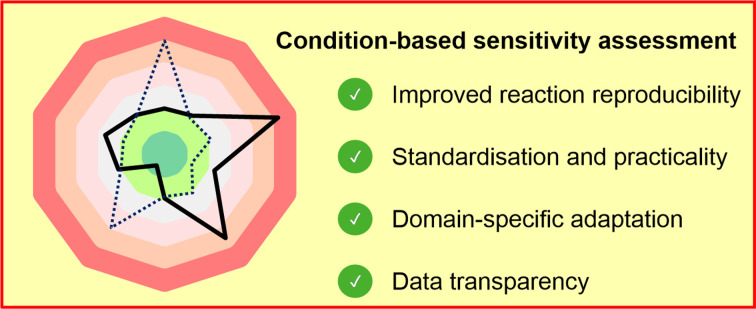

Reproducibility and practicality of synthetic protocols form fundamental pillars within the realm of experimental science.^1^ From basic determinations, like selecting a synthetic procedure for starting materials, to strategic judgments such as project viability and the evaluation of scientific reports for publication or funding decisions, chemists depend on the insights provided within scientific literature. Understanding a reaction's sensitivity towards a diverse set of parameters is a key factor that ultimately leads to more sustainable and affordable chemistry,^2^ enabling more robust^3^ and greener reactions.^4^

The range of reactions utilised in synthetic disciplines like medicinal or process chemistry has seen little expansion, despite organic chemists consistently devising more efficient, selective, and innovative methods.^5^ Particularly, technologies often found in academic research, like photo- and electrochemistry, are not frequently employed in complex syntheses or medicinal chemistry projects. Scaling up reactions is a common task in the chemical industry, while miniaturisation of chemical reactions smaller than milligram scale represents an entirely different challenge.^6^ As high-throughput experimentation (HTE) becomes increasingly important, alongside advancements in data science and molecular machine learning, ensuring the quantitative accuracy of chemical reactions becomes increasingly vital for building reliable datasets.^7–9^

While the general outcome of chemical reactions tends to align with existing literature, there is often variability in quantitative results such as yield, selectivity, and purity. Systematically capturing these variations helps in integrating chemical literature into practical industrial processes and application across different research domains and academic labs. Even with detailed protocols provided in ESI,† reaction parameters may differ between labs with different chemists conducting the experiments and assessing this impact on the target value (*e.g.* yield) is non-trivial.

Depending on the resources, data and time available, a chemist may choose between different approaches in determining the sensitivity of a process or reaction. Recently, data science methods like molecular machine learning have emerged as rapid and cost-effective tools for predicting yields, but they rely on dense and unbiased datasets, which are often unavailable.^8,10^ Conducting Design of Experiment (DoE) studies to explore every aspect of a reaction reveals its sensitivity, but this approach is likely the most time-consuming and costly.

Convenient experimental sensitivity assessment tools offer an advantageous alternative, providing researchers with efficient means to evaluate the response of chemical processes to various factors. These tools not only streamline the analysis process but also empower scientists to swiftly identify critical parameters influencing reactions, thereby facilitating more informed decision-making in experimental design and optimisation (Fig. 1).

**Fig. 1:**
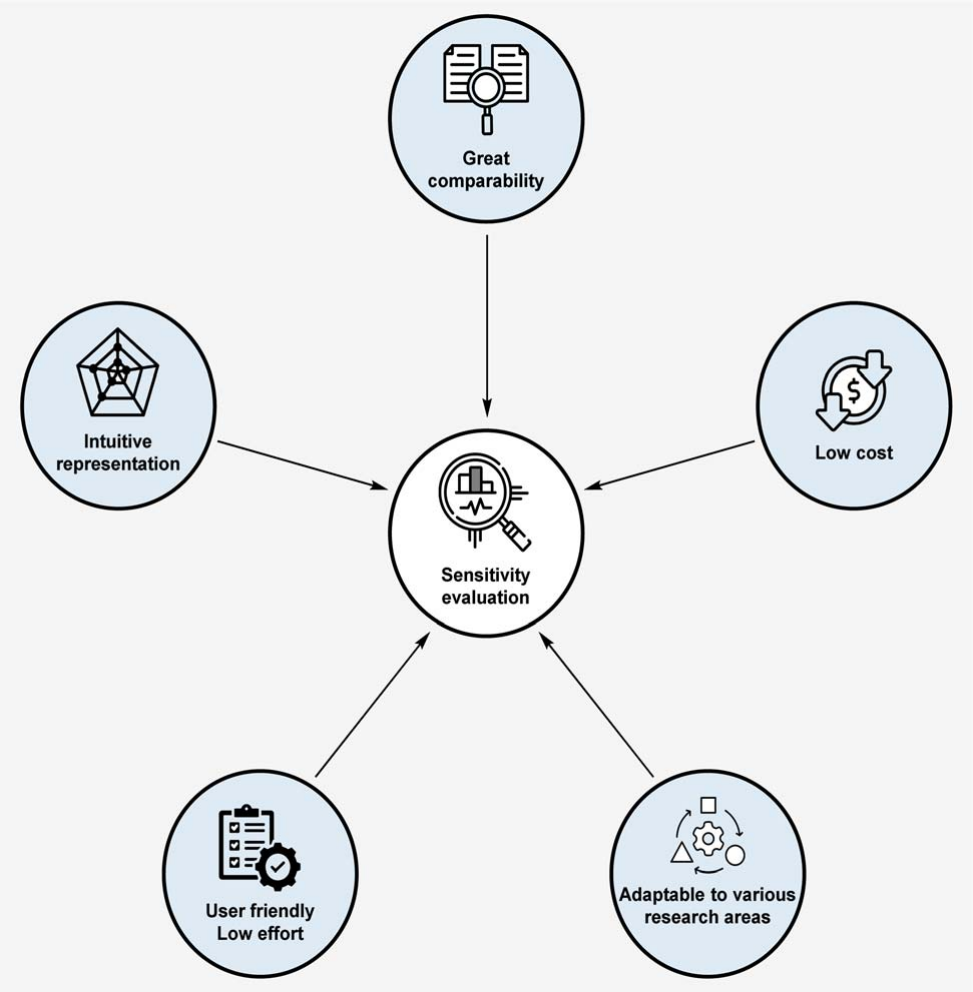
Guidelines for conducting a universally applicable sensitivity assessment of reaction conditions.

In response to this challenge, the sensitivity screen was developed as a general tool to tackle the aforementioned reproducibility problems.^11^ In this intuitive graphical evaluation, the effect of the parameters is plotted on a radar diagram, showing their deviation from the target value (usually yield) compared to standard conditions. An updated version of the sensitivity screen with improved design and the option to use an additional target value (*e.g.* selectivity), together with a detailed user guide is provided in the ESI.† Our own experience showed that, if a synthetic protocol cannot be reproduced immediately, the vast number of possible sources of error is often overwhelming and thus troubleshooting can be incredibly time consuming. This especially applied to photochemical methods, as this rapidly developing field suffers from many different experimental setups, causing reproducibility issues. The basic idea to solve this issue was to use a screening approach to identify the crucial reaction parameters, which most significantly influence the reaction's behaviour. To do so, single reaction parameters are varied in both positive and negative directions, while all other parameters are kept constant. The resulting impact on the yield provides a valuable clue regarding which parameters to prioritize when troubleshooting reproducibility issues. To ensure user-friendliness, the method was designed to work with standard equipment and a stock solution approach, to help minimise experimental effort. As an intuitive graphical representation of the respective results, we introduced a colour-coded radar/spider diagram, visualising the results and highlighting the crucial parameters.

Our innovation was embraced by the scientific community and has since been widely adopted for applications beyond the originally suggested ones. Originating from a background in photochemistry, we initially considered only yield as the target value and parameters such as: temperature, concentration, oxygen and moisture levels, light intensity, and scale. The use of the sensitivity screen within the scientific community, including our own group, has led to its adoption modified with additional parameters and target values. While the target value was formerly only yield, researchers have suggested and applied conversion, product ratio, selectivity, ee, throughput, TON, radiochemical yield (RCY), molar/specific activity (*A*_m_/*A*_s_), *H*/*D*-ratio, purity, space-time yield and enzyme activity,^12^ and the standardisation of these terms has been a central achievement of the chemical community (Fig. 2).^13–16^ Especially for industrial processes with more than one desired product, the reaction can be steered towards a customised product distribution, which might alter given the demand of the chemical product. A radar diagram might also be beneficial for straightforward product ratio adjustment.

**Fig. 2:**
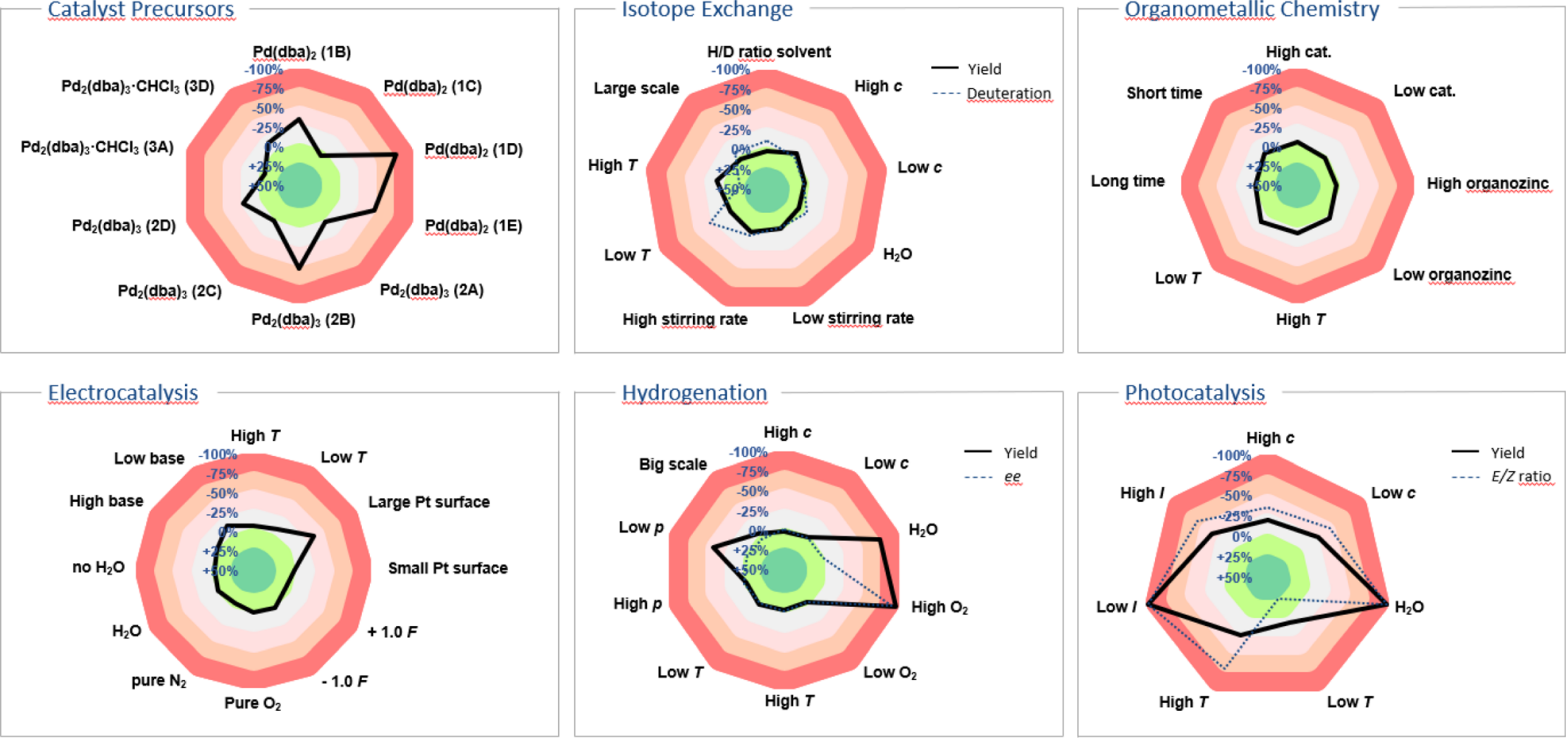
Examples showcasing sensitivity screens tailored to diverse research fields.^13–16^ In instances like hydrogenation, isotope exchange and photocatalysis the sensitivity screen was utilised to illustrate two target values, such as yield/deuterium incorporation, yield/enantiomeric excess and yield/*E*:*Z* ratio, along with the parameters' impact on them.

We have been pleasantly surprised by the diverse applications of the sensitivity screen, which have both refined the original parameter choices made by other scientists and extended its utility into various fields such as electrochemistry, hydrogenation, isotope chemistry, and asymmetric synthesis, among others, over the past five years.

In some cases, the critical adjustment of parameter values served as valuable feedback on the sensitivity screen: we acknowledge that our proposed range of ±10% concentration was frequently insufficient for detecting substantial yield changes beyond the detection limit of the analytical method employed. The widespread adoption of at least a ±50% concentration variation by many groups to demonstrate the impact on the target value reflects the valuable feedback from scientists in using our screening method.

For the other variables, we want to emphasise that our initial sensitivity screen was not intended to function as a comprehensive review of control reactions, where parameters should be varied significantly to demonstrate drastic yield changes. Instead, its purpose is to illustrate how minor alterations in various laboratory settings and applications can lead to significant yield variations, such as impurities like the presence of water in solvents and substrates, or minor oxygen ingress.

We continue to value the utilisation of the radar diagram depiction and sensitivity approach by all scientists, recognising that alternative and unconventional applications of this tool contribute significantly to collective efforts aimed at enhancing the reproducibility of reactions within the scientific community.

Reviewing and exploring the diverse application of the sensitivity screen across various domains of chemical synthesis and research, we have compiled a summary of its utilisation by various academic and industrial laboratories. Drawing inspiration from the extensive adaptations since our initial publication, we have included additional recommendations aimed at implementing the utilisation of sensitivity-based assessments across diverse research fields (Fig. 3).

**Fig. 3:**
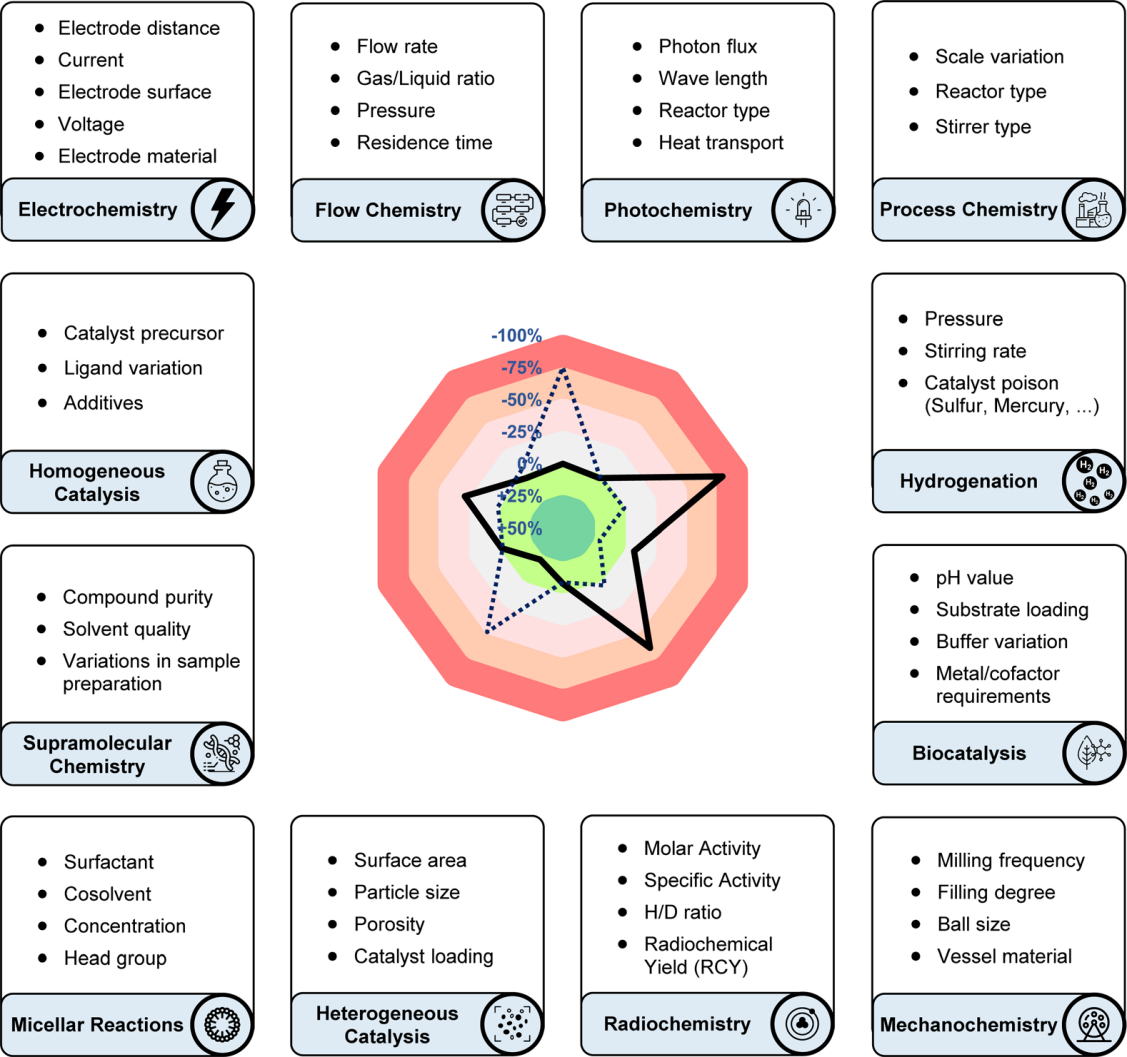
Suggestions for parameters to include in a sensitivity screen across various research domains. While being established in some fields (hydrogenation, photochemistry, electrochemistry), additional recommendations for the application of the sensitivity screen in flow chemistry, mechanochemistry and biocatalysis aim to inspire scientists from these areas.

## General parameters

While variations in stoichiometry are typically addressed in optimisation tables, the sensitivity screen offers a rapid assessment of their significance, particularly in multicomponent reactions.^17^ Catalyst loading and the quantity of additives are intriguing parameters pertinent to scaling up processes and may have to be varied significantly depending on the intended application. Frequently neglected parameters in published laboratory procedures encompass stirring rate, reaction duration (including “overnight” reactions), and the purity of substrates and reagents.

Bull and co-workers introduced substrate purity as a new parameter.^18^ They repurified their carboxylic acid starting material, enhancing its purity from 98.9% to 99.9%. This small adjustment, as revealed by their sensitivity screen, led to a notable +23% increase in yield, highlighting the significant impact even minor parameter changes can have. When Baran and co-workers established new anomeric amide reagents for electrophilic halogenation, they could demonstrate that these are very insensitive to variations in reaction parameters, even water and air intake, thus confirming their practicality.^19^

### Electrochemistry

Electrochemical synthesis has experienced a renaissance in its use as a tool in organic chemistry, showcasing multifaceted utility. The availability of standardised commercial electrochemistry equipment, coupled with detailed reaction protocols, has significantly contributed to enhancing reproducibility in this field. Nevertheless, the myriad of additional parameters associated with electrochemical processes, such as variations in electrode surface area, materials, distances, supporting electrolytes, potential, or current, pose challenges in discerning significant deviations from the original procedure and those deemed negligible. Hilt and colleagues have effectively applied a sensitivity-based assessment in their methodology papers, leading to the conclusion that larger electrode surfaces significantly decrease the yield of their protocols while a smaller electrode distance can have variable effects, depending on the specific reaction system.^14,20^ The use of alternating current/polarity instead of traditional direct current deserves special attention^21^ since the nature of the alternating current can be widely varied in terms of frequency and nature of the potential curve, and future electrochemical methodologies might even use pulsating or variable current, parameters that can be included in a sensitivity screen.

### Flow chemistry

Flow chemistry not only facilitates the continuous production of chemical reactions but also serves as an enabling technology for the transition and upscaling of newly developed methodologies, such as photochemistry and gas–liquid reactions.^22^ Key parameters in flow chemistry, including flow rate, residence time, gas/liquid ratio, and pressure, play pivotal roles in process optimisation and control.

### Photochemistry

Coming from a background rooted in photochemistry, our initial publication on sensitivity screening primarily focused on photochemical transformations and their corresponding parameters. Since its inception, it has been warmly embraced by the photochemical community, finding applications spanning from metallaphotoredox^23^ to direct sensitisation^24^ and energy transfer catalysis.^25^ However, the quest for reproducibility^26^ and comparative analysis in the sensitivity of photochemical reactions remains a daunting challenge. As a result, several notable approaches and critical evaluations have emerged from the scientific community. Ziegenbalg and colleagues, for instance, meticulously categorised parameters sorted by how commonly they are studied in publications, highlighting crucial factors such as reactor type, photocatalyst counter anion, heat and mass transport, temperature effects, reactor operation mode, and photon flux—some of which are often overlooked or inadequately explored.^27^ Specifically, heat transport was found to play a crucial role and can dramatically change reaction outcomes when reactions are scaled up. In addition, several studies have examined and compared the design and use of commercial photoreactors, highlighting differences in irradiation uniformity and temperature control between different setups.^7,28^ Despite these insightful analyses, the application of photocatalysis must navigate potential pitfalls in technology transfer,^29^ particularly concerning the scalability of reactions.

### Micellar reactions

The evolution of organic solvent selection in chemical processes reflects ongoing efforts to enhance reaction efficiency and sustainability.^30^ This development has sparked widespread interest in alternative reaction media, with water emerging as a particularly compelling option.^31^ Micellar catalysis and reactions have paved the way for exploring water as a promising alternative reaction medium.^32^ Its use and optimisation introduces the key parameters surfactant and cosolvent, and collaborative studies have shown good reproducibility for several reaction classes.^33–36^ We propose that conducting sensitivity assessments on these reactions, comparing different surfactants and conditions, will accelerate and simplify their industrial and academic adoption, especially since pioneering reactivity approaches on oil–water interfaces are on the rise.^37^

### Mechanochemistry

Mechanochemistry shares closer ties with traditional thermal energy input methods compared to for example photo- or electrochemistry. Various parameters such as milling frequency, ball size, filling degree, and even the material composition of the vessel and balls can significantly influence reaction outcomes.^38^

### Radiochemistry and isotope labelling

Radiochemical yield (RCY), molar activity (*A*_m_) and specific activity (*A*_s_)^39^ could all be potential target values in sensitivity screen assessments for radiochemical reactions. Given the short half-life of many isotopes, the duration of reaction time becomes paramount in these instances, with sensitivity towards the timing of individual steps assisting scientists in optimising radiochemical yield across consecutive stages. Van Gemmeren and colleagues introduced solvent *H*/*D* ratio as a novel reaction parameter for β-C(sp^3^)–H deuteration of free carboxylic acids, showcasing their reaction's adaptability to partially deuterated solvents.^15^ In their sensitivity assessment, they combined target values for both product *H*/*D* ratio and yield, visually capturing the optimal compromise temperature in the corresponding radar diagram (Fig. 2).

### Supramolecular chemistry

Meijer and co-workers have aptly summarised the most prevalent reproducibility issues in supramolecular chemistry, namely:^40^ compound purity, solvent quality, and variations in sample preparation, such as slight temperature fluctuations or differing ramping speeds. Considering the sensitivity of many supramolecular applications to these minor changes, which may not necessarily affect the outcome measurably, we anticipate that the sensitivity screen will be beneficial but requires careful calibration for both the synthetic assembly of supramolecular complexes and their application.

### Biocatalysis

Drawing parallels with the utilisation of additive-based robustness screens in biochemical contexts,^41,42^ we anticipate significant potential for employing the sensitivity screen in chemical reactions being applied to biocatalysis, proximity labelling, and bioorthogonal chemistry settings.^43^ Given the inherently higher complexity of biological systems and enzyme activity, we suggest incorporating: variations in buffer composition, pH sensitivity, metal/cofactor requirements, and substrate loading into a biochemical sensitivity screen. Successful transition from chemical methodology based screening approaches to other life sciences has so far found widespread use by the scientific community,^41,44^ and the sensitivity screen has the potential to follow up on these examples.

### Hydrogenation

Hydrogenation reactions are some of the most widely used transformations, reflected by the fact that 25% percent of all chemical value chains in industry include at least one hydrogenation step.^45^ The hydrogen mass transport in between the different phases (gas–liquid, liquid–solid) has a pronounced impact on the rate and outcome of the reaction. Stirring rate as a single parameter has been shown to influence both yield^46^ and enantioselectivity,^47^ while in other cases oxygen had no effect on yield but severely lowered the *ee*.^48^ Hydrogen pressure alters the concentration of dissolved hydrogen in the liquid phase, resulting in a higher reaction rate and potentially lower selectivity.^49^ Catalyst poisoning can also be a serious problem, especially for industrial processes *e.g.* oil refining, and is the main reason why hydrodesulphurisation is often necessary beforehand.^50^ Consequently, catalytic systems resistant to small amounts of poison are sought after,^51^ and evaluating the poisoning sensitivity as a parameter represents vital information for synthetic chemists.

### Homogeneous catalysis and cross-coupling reactions

Similarly, in homogeneous catalysis, variations in catalyst properties and quality can be substantial, especially when metal–ligand complexes are formed *in situ*. Gooßen and colleagues investigated the impact of different commercial palladium precatalyst batches and found considerable yield discrepancies among batches, with no single physical or spectroscopic descriptor offering reliable discrimination.^16^ A depiction of parts of their study is shown utilising a sensitivity radar diagram in Fig. 2, demonstrating the significant influence of precatalyst selection on the Buchwald–Hartwig amination reaction.

### Heterogeneous catalysis and surface chemistry

Given the inherently complex and unpredictable nature of heterogeneous catalytic reactions, sensitivity assessments of these reactions require careful consideration, particularly regarding the impact of reaction parameters. Catalyst samples can exhibit significant variations in particle size and properties. While precise control over these parameters may not always be feasible, conducting structure–activity relationship studies alongside sensitivity-based assessments, encompassing batches with diverse properties or from different manufacturers, facilitates straightforward comparisons of these parameters. These may encompass catalyst loading, porosity, surface area, particle size, and susceptibility to common catalyst poisons introduced by substrate impurities, particularly sulphur-containing compounds. Initiatives like rigour and reproducibility (R&R), in which Schweitzer and colleagues share best practices on reproducibility in thermal heterogeneous catalysis, are crucial, subject-specific and orthogonal approaches to the use of the sensitivity screen.^52^

### Process chemistry

For industrial processes involving the simultaneous synthesis of multiple desired products, the reaction can be directed towards a desired product ratio, which may change based on the demand for the chemical product. In such cases, the operator might need to make adjustments, and a sensitivity screen can be useful for facilitating this process, allowing a straightforward visualisation of how parameters effect the new target value. The changes in overall dimensions resulting from variations in pilot scale, reactor type, and adjustable parameters can be assessed through sensitivity evaluation. For example, this assessment may involve comparing different types of stirrers, such as paddle, tooth, anchor, frame, ribbon screw, and turbine designs.^53^ Analysing the characteristics of multiphase reactors, Papayannakos and co-workers have used the ratio of gas to liquid velocities as the central parameter across different reactors, providing a key feature for a chemical engineering approach towards sensitivity assessment.^54^

## Conclusion and outlook

Sensitivity evaluation offers a rapid, cost-effective method for reaction evaluation, aiding in the adoption and implementation of newly developed methodologies by providing chemists and scientists from diverse fields with an overview of parameter impacts. The preceding discussion and suggestions regarding the versatile application of novel parameters in assessments across various domains are intended to inspire researchers in these fields to utilise the sensitivity screen. In doing so, they can shed light on specific challenges related to optimisation and sensitivity within their respective fields, thus providing valuable insights for the wider scientific community.

We want to emphasise that the sensitivity screen is a valuable and economic tool, but for an in-depth evaluation of reactions it should be followed up by more detailed approaches such as Design of experiments (DoE), mechanistic analysis, and advanced process optimisation. It serves as an initial, cost-effective and quick guide for chemists in these endeavours. Although standardised sensitivity assessments are desirable, the need for customisation of parameters specific to each field can hinder comparability, especially when the chemistry deviates significantly from traditional batch processes.

Our sensitivity screen has been widely embraced by chemists across various disciplines, generating a comprehensive dataset of sensitivity information. This data serves not only as a reference tool for researchers but also holds potential for informing data-driven models for sensitivity prediction in the future. We strongly believe that broader application across multiple fields will help advance the common goal of increasing transparency in newly published research and enhancing reproducibility within academia and beyond.

## Author contributions

All authors contributed to the conceptualization and literature search, wrote the manuscript and were involved in revising, editing, and proofreading.

## Conflicts of interest

There are no conflicts to declare.

## Supplementary Material

SC-015-D4SC03017F-s001

## Data Availability

This is a perspective manuscript and the original data is publicly available and can be found in the original manuscripts.
